# COVID-19–Related Infodemic and Its Impact on Public Health: A Global Social Media Analysis

**DOI:** 10.4269/ajtmh.20-0812

**Published:** 2020-08-10

**Authors:** Md Saiful Islam, Tonmoy Sarkar, Sazzad Hossain Khan, Abu-Hena Mostofa Kamal, S. M. Murshid Hasan, Alamgir Kabir, Dalia Yeasmin, Mohammad Ariful Islam, Kamal Ibne Amin Chowdhury, Kazi Selim Anwar, Abrar Ahmad Chughtai, Holly Seale

**Affiliations:** 1 Program for Emerging Infections, Infectious Diseases Division, icddr,b, Dhaka, Bangladesh;; 2 School of Public Health and Community Medicine, University of New South Wales, Sydney, Australia;; 3 Khulna University of Engineering and Technology, Khulna, Bangladesh;; 4 Mahidol University, Nakhon Pathom, Thailand;; 5 Centre for Primary Health Care and Equity, University of New South Wales, Sydney, Australia;; 6 Department of Infectious Diseases and Microbiology, School of Medicine, International University of Health and Welfare, Narita, Japan

## Abstract

Infodemics, often including rumors, stigma, and conspiracy theories, have been common during the COVID-19 pandemic. Monitoring social media data has been identified as the best method for tracking rumors in real time and as a possible way to dispel misinformation and reduce stigma. However, the detection, assessment, and response to rumors, stigma, and conspiracy theories in real time are a challenge. Therefore, we followed and examined COVID-19–related rumors, stigma, and conspiracy theories circulating on online platforms, including fact-checking agency websites, Facebook, Twitter, and online newspapers, and their impacts on public health. Information was extracted between December 31, 2019 and April 5, 2020, and descriptively analyzed. We performed a content analysis of the news articles to compare and contrast data collected from other sources. We identified 2,311 reports of rumors, stigma, and conspiracy theories in 25 languages from 87 countries. Claims were related to illness, transmission and mortality (24%), control measures (21%), treatment and cure (19%), cause of disease including the origin (15%), violence (1%), and miscellaneous (20%). Of the 2,276 reports for which text ratings were available, 1,856 claims were false (82%). Misinformation fueled by rumors, stigma, and conspiracy theories can have potentially serious implications on the individual and community if prioritized over evidence-based guidelines. Health agencies must track misinformation associated with the COVID-19 in real time, and engage local communities and government stakeholders to debunk misinformation.

## INTRODUCTION

The term infodemic, defined as “an overabundance of information—some accurate and some not—that makes it hard for people to find trustworthy sources and reliable guidance when they need it,” was coined to categorize some of the common features of rumors, stigma, and conspiracy theories during public health emergencies.^1^ During the Ebola outbreak in the Democratic Republic of Congo in 2019, misinformation was linked to violence, mistrust, social disturbances, and targeted attacks on healthcare providers.^2^ During the SARS outbreak in China in 2002–2003, fear and anxiety about contracting the disease caused social stigma against Asian people.^3^ Stigmatized persons may delay seeking medical care, potentially remaining undetected, but contributing to the expansion of the epidemic via community transmission.^4,5^ The UN secretary-general identified COVID-19–related rumors as a global enemy.^6^ Globally, there have been reports of rumors, stigma, and conspiracy theories connected to the ongoing COVID-19 pandemic.^7^

Public health emergencies are stressful times for people and communities.^8^ Managing rumors, dispelling misinformation and conspiracy theories, and mitigating fear and stigma directed toward persons and places affected are essential to pandemic preparedness and control.^3^ International health agencies, including the WHO, recognized rumor, stigma, and conspiracy theories as emerging threats to pandemic preparedness and control, and, therefore, recommended systematic monitoring and control measures.^9,10^

For novel epidemic and pandemic diseases, the detection, assessment, and response to rumors, stigma, and conspiracy theories and their impact on public health in real time are a challenge.

Facebook, Twitter, and online newspapers have been identified as the best platforms for monitoring misinformation and dispelling rumors, stigma, and conspiracy theories among the general people.^11^ Epidemiological monitoring on online media platforms includes extracting, aggregating, analyzing online textual data in real time, and devising control measures.^12^ Although social media data are observational, they can complement traditional surveillance system data.^13^ Since the onset of the COVID-19 pandemic, social media users have been playing a role in all stages of knowledge translation, including COVID-19 morbidity and mortality, interventions, spreading rumors and conspiracy theories, and reporting stigma. Therefore, we followed and examined COVID-19–related rumors, stigma, and conspiracy theories circulating on online platforms, including fact-checking agency websites, Facebook, Twitter, and online newspapers, and their impacts on public health.

## METHODS

### Study settings and data collection.

We formed a team of social scientists, medical doctors, and epidemiologists to collect and review infodemics. The team retrospectively collected COVID-19–related infodemic reports between December 31, 2019, when the WHO China office was notified about the outbreak, and April 5, 2020. The team reviewed a wide range of sources, including fact-checking agency websites, Facebook, Twitter, websites for television networks, and newspapers. To increase the scope of data collection, the team also subscribed to websites of different national and international television networks and newspapers, national and international fact-checking agencies, the WHO, and U.S. CDC. Most of the retrieved items were English-based media sources; however, the items in other languages were translated using Google translator.

### Study definitions.

Rumor is defined as unverified information that can be found as true, fabricated, or entirely false after verification.^14^ Stigma is a socially constructed process through which a person with stigma can experience discrimination and devaluation in society.^15^ A conspiracy theory is defined as explanatory beliefs about an individual or group of people working in secret to reaching malicious goals.^16^

### Data extraction and consistency.

The team reviewed the reports collected on rumors, stigma, and conspiracy theories, and entered the data into a Google spreadsheet. The exclusion criteria included duplicate contents and contents that could not be translated into English. The use of a password-protected Google sheet avoided duplication, and data from each information source were double-checked; 5% of the data were further checked for consistency. Any discrepancies between reviewers were resolved by discussion with the lead author.

### Analysis.

We analyzed the data using the open-source statistical package R version 3.6.3 (R Foundation for statistical computing, Vienna, Austria, Available at: https://www.R-project.org/). We undertook a descriptive analysis of the quantitative data. We used stacked bar charts to depict the distribution of different infodemic categories by date and country. We constructed a global map to examine the spatial distribution of the total count of the rumors, stigma, and conspiracy theories reported. For newspaper and online articles, content analysis was undertaken of the news articles to compare and contrast the data collected. Based on the definition provided in Table 1, data were reviewed and categorized into three categories: rumors, stigma, and conspiracy theories. We then used four themes that WHO predefined,^17^ including the cause of disease, illness, treatment, and interventions. After reviewing the data, we added another theme, violence. The coded data were then shared with the team for review and consensus. The team also categorized the rumors as true, false, misleading, and not proven.

**Table 1:** Operational definitions used in the study

Categories	Operational definitions
Rumor	Rumor was defined as any unverified and instrumentally relevant claims, statements, and discussion centering COVID-19 circulated in online platforms.
Stigma and discrimination	We defined stigma as a socially constructed phenomenon through which a person is directly or indirectly labeled by their illness, exposures, travel history, and ethic descents that further led to negative actions and discrimination.
Conspiracy theory	Statements, claims, and discussion of various theories related to the origin of SARS-CoV-2 and its malicious goals.
These aforementioned types of infodemic were further classified in the following categories.	
Cause of the disease	How did the SARS-CoV-2 emerge and what is the reason?
Illness	Statements, claims, and discussion around signs and symptoms of COVID-19, its transmission dynamics, and mortality.
Treatment	Statements, claims, and discussion about diagnostic tests, home remedies, and traditional medicines as a cure of COVID-19.
Interventions	Steps taken by the health authorities, governments, or other allied institutions to prevent transmission of COVID-19.
Violence	Physical or verbal assaults toward any person in community or in the workplace.
Miscellaneous	Statements, claims, and discussion that did not fit into said categories.

## RESULTS

**Figure 1.**
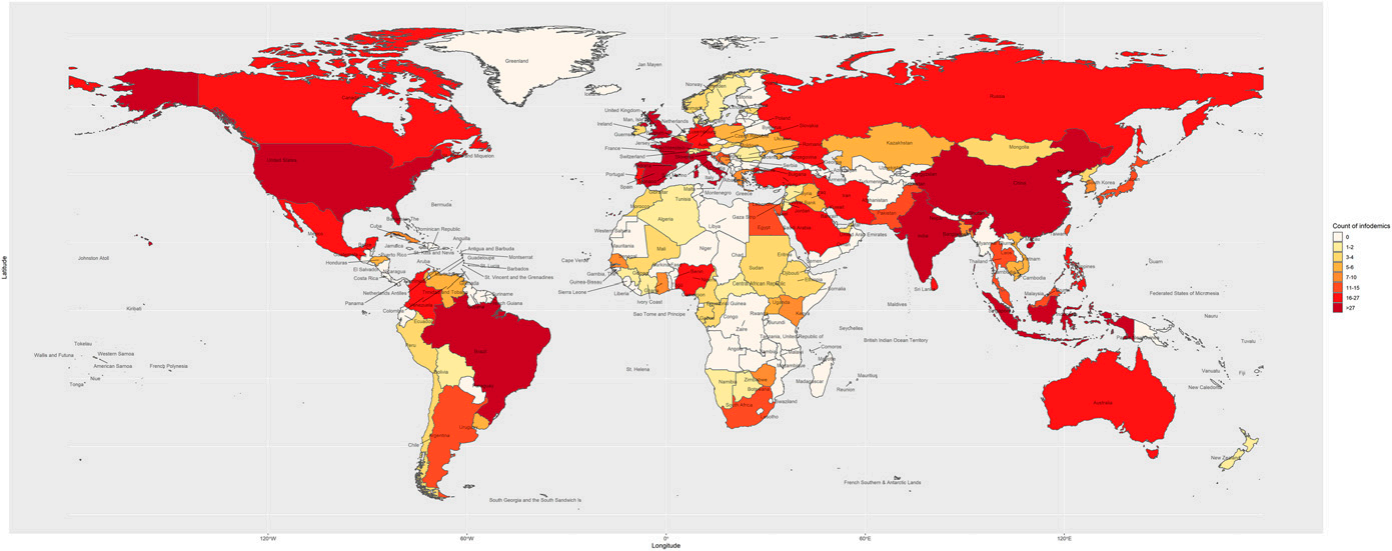
Distribution of rumor, stigma, and conspiracy theories related to COVID-19 identified during the study, 2020.

**Figure 2.**
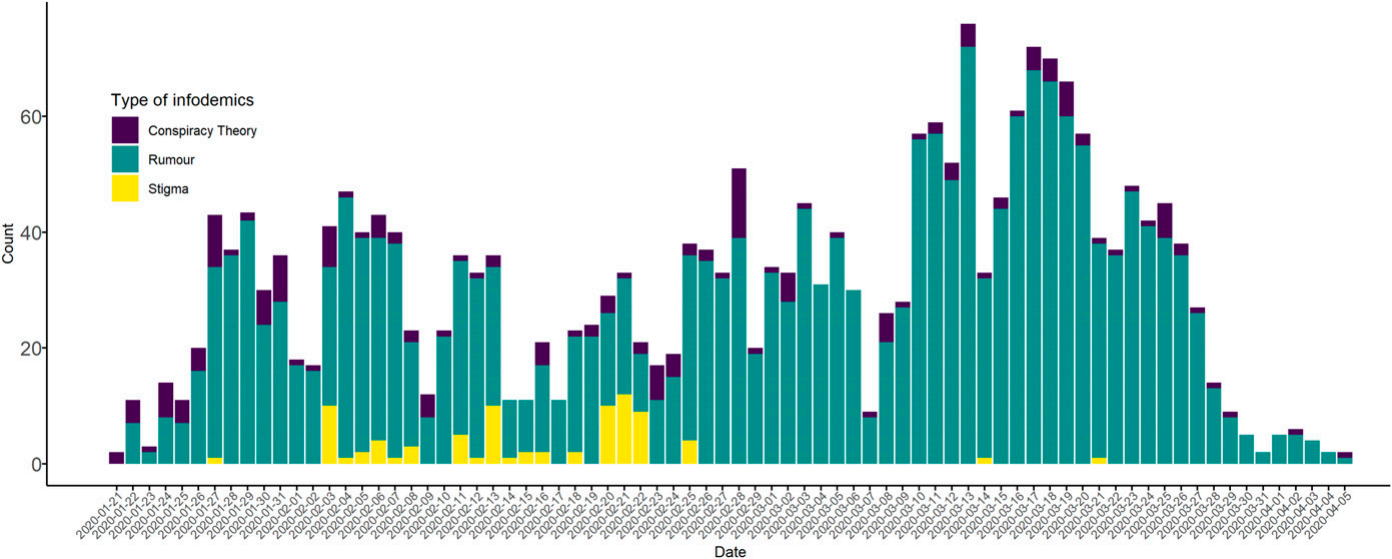
Rumors, stigma, and conspiracy theories associated with COVID-19 and time line of reports detected during the study, 2020.

We identified 2,311 reports related to COVID-19 infodemic in 25 languages from 87 countries (Figure 1). Of these, 2,049 (89%) of the reports were classified as rumors, 182 (7.8%) were conspiracy theories, and 82 (3.5%) were stigma. The study identified three waves of infodemics between January 21, 2020 and April 5, 2020 (Figure 2). The first wave was between January 21 and February 13, the second wave was between February 14 and March 7, and the third wave was between March 8 and March 31, 2020. In the first two waves, the numbers of reported infodemics were low, and the pattern was similar when compared with that of the third wave.

**Figure 3.**
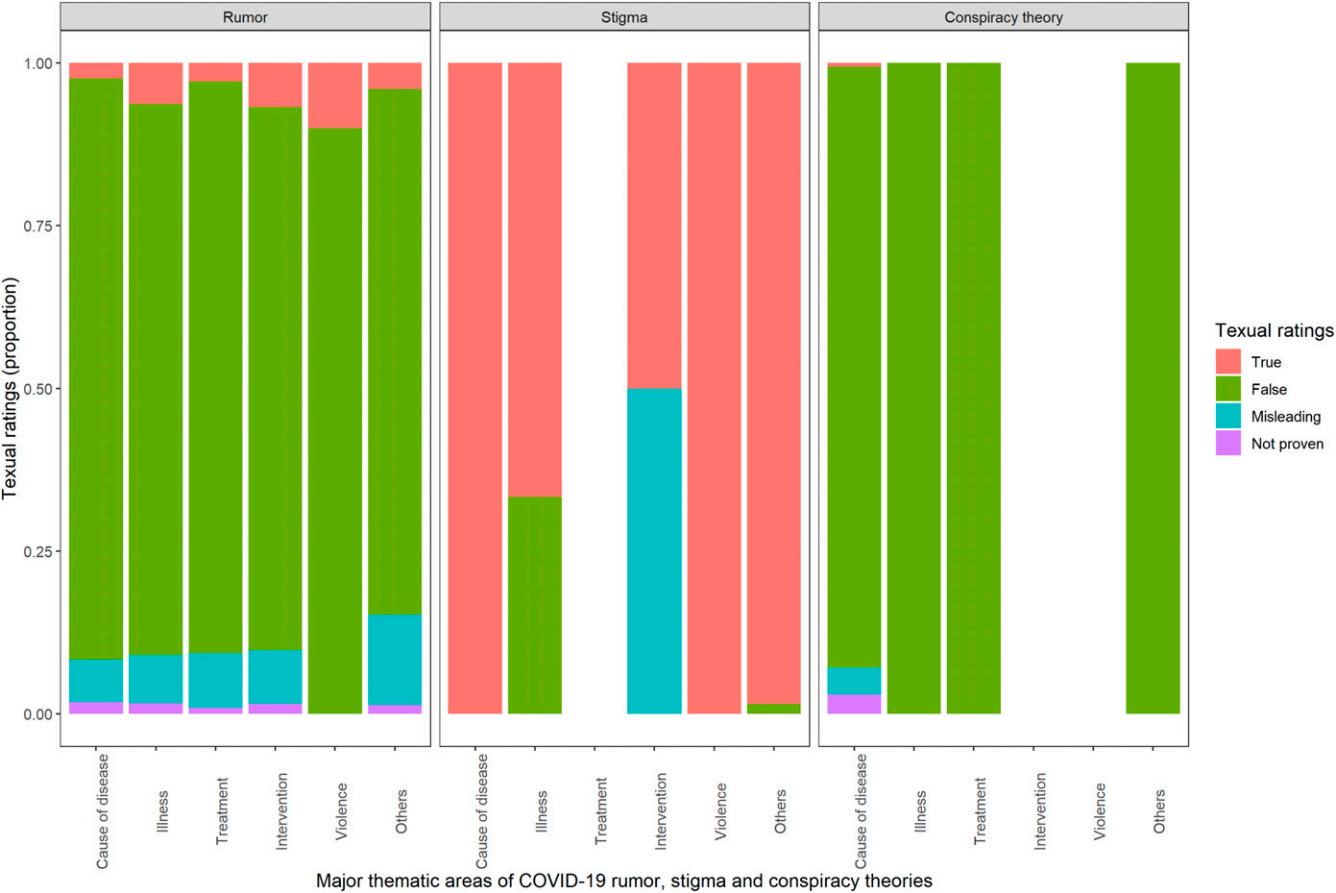
Factual accuracy of rumors, stigma, and conspiracy theories linked to COVID-19 causes of disease, illness, treatment, and control measures.

**Figure 4.**
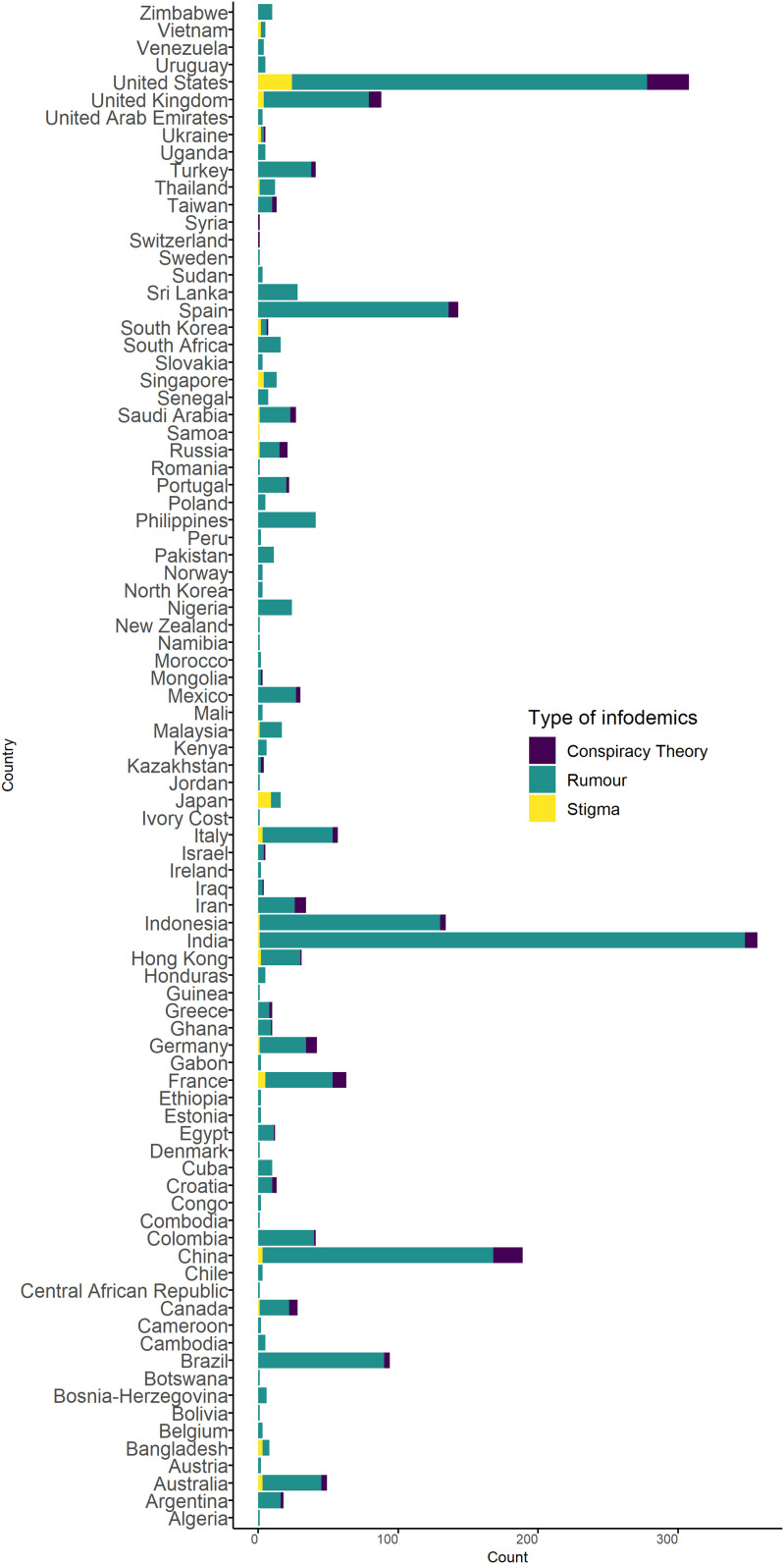
Rumors, stigma, and conspiracy theories related to COVID-19 by countries detected during the study, 2020.

Among all the categories of information tracked, 24% were related to illness, transmission, and mortality; 21% to control interventions; 19% to treatment and cure; 15% to the cause of disease including the origin; 1% to violence; and 20% to miscellaneous. Of the 2,276 reports for which text ratings were available, 1,856 claims were false (82%), 204 were correct (9%), 176 were misleading (8%), and 31 were not proven (1%) (Figure 3). Most of the rumors, stigma, and conspiracy theories were identified from India, the United States, China, Spain, Indonesia, and Brazil (Figure 4).

### Rumors.

Among all categories of infodemics we tracked, rumor was the most prevalent. The volume of rumors increased from February and continued until the end of the study period, peaking in the middle of March 2020 (Figure 2). Most of the rumors were related to COVID-19 illness, transmission, and mortality, followed by interventions focusing on infection prevention and control measures. There were reports about eating garlic, keeping the throat moist, the need to avoid spicy food, and the importance of taking vitamins C and D to help prevent the disease (Table 2). Moreover, there were reports of rumors that spraying chlorine could prevent coronavirus infection.

**Table 2:** Rumor, stigma, and conspiracy theories related to COVID-19 in the globe, 2020

Rumor
“Novel coronavirus is in the cloud”
“Coronavirus is a snake flu”
“Pet animals are the sources of coronavirus”
“Novel coronavirus strain is a type of rabies”
“Coronavirus outbreak in the livestock”
“Poultry eggs are contaminated with coronavirus”
“Cookies, rice, and Chinese red bull were contaminated with the virus”
“Eating bat soup is the source of the (COVID-19) outbreak”
“COVID-19 found in orange”
“Coronavirus from imported goods”
“Mobile phone can transmit coronavirus”
“Notes are sources of coronavirus”
“Common cold had been renamed as coronavirus”
Rumor about treatment, prevention, and control
“Eating garlic can cure coronavirus”
“Drinking bleach may kill the virus”
“Drinking alcohol may kill the virus”
“Gargling vinegar and rose water or vinegar and salt may kill the virus in throat”
“Drinking cow urine and cow dung can cure coronavirus”
“Silver solution for coronavirus treatment”
“Wearing warm socks, mustard patches, and spreading goose fat on one’s chest as treatment”
“Keeping throat moist, avoid spicy food and taking vitamin C may prevent the disease”
“Avoiding cold or preserved food and drinks, such as ice cream and milkshakes may prevent infection”
“Spraying chlorine all over your body can prevent coronavirus infection”
“Sesame oil can prevent coronavirus infection”
“Granite bath can prevent coronavirus infection”
“Sea lettuce can prevent coronavirus infection”
“Vitamin C intake can prevent coronavirus infection”
“Vitamin D can prevent coronavirus infection”
“Eating *Centella asiatica* may prevent coronavirus infection”
“Drinks containing mint or white willow, and spices like saffron, turmeric, and cinnamon would strengthen the lungs and the immune system against the virus”
“Rinse mouths with salt water solution to prevent infection from the new virus outbreak”
“Do not hold your thirst because once your membrane in your throat is dried, the virus will invade into your body within 10 minutes”
“Applying petroleum jelly around your nostrils will protect against dangerous air pollutants”
“Do-it-yourself coronavirus detection test”
“Cannabis boosts immunity against the novel coronavirus”
“Frequent washing clothes can reduce transmission”
Conspiracy theory
“Novel coronavirus is engineered, laboratory-generated virus either accidentally or deliberately released in the area of the Wuhan seafood and animal market”
“COVID-2019 outbreak was planned”
“It’s a bio-weapon funded by the Bill & Melinda Gates foundation to further vaccine sales”
“Biological weapon manufactured by CIA”
“President Donald Trump targeted the city with coronavirus to damage its culture and honor in Iran”
“The virus is an attempt to wage ‘economic war on China’”
“America’s Jews are driving America’s wars”
“This outbreak is a medical terrorism”
“Zionists are against regional security”
United States and Israel of being behind the creation and spread of the deadly coronavirus as part of an economic and psychological war against China
“This outbreak is a population control scheme”
“Tom Cotton claimed that COVID-19 was manufactured in Chinese bio-laboratory”
“Rush Limbaugh opining that whole COVID-19 is a conspiracy against Trump to let him down in election. He purported it as a worse flu”
“New coronavirus vaccines already exist”
“Pneumonia vaccines are effective against the Wuhan coronavirus”
“Israel has sent a vaccine to Wuhan city for patients infected with coronavirus”
Stigma
“I am not a virus: French Asians angered by racism”
“Chinese are uncivilized”
“Chinese are bioterrorists”
“A French newspaper with headline ‘Yellow Alert’ tagged a Asian woman image wearing mask”
“Chinese are dropping their coronavirus”
“Every disease has ever came from China”
“Keep your virus, dirty Chinese”
“Chinese dietary habit caused COVID-19”

In addition to food and vitamins to boost up immunity, some reports focused on the so-called treatments such as miracle mineral solutions that involved mixing sodium chlorite solution with citric acid^18,19^ or drinking bleach or alcohol for immunity and cures.^17^ Other reported rumors related to cures were drinking tea and cow urine or dung in India,^20^ camel urine with lime in Saudi Arabia, and medicinal plants in Africa.^21^ Moreover, information on how to self-diagnose coronavirus was muddled by unverified sources. For example, holding one’s breath for more than 10 seconds could help self-diagnose coronavirus infection was a popular circulating myth.^22^

### Stigma.

In several countries, people, including healthcare workers, were bullied or physically insulted or faced discrimination from their landlords and neighbors. According to a doctor’s interview in the *Guardian*,^23^ “Quite a few doctors have decided to spend the next few days in the restrooms of hospitals because they have lost their apartments or could not get into the apartments because of the hostility from the people of their community.” In Australia, a medical health worker with Chinese heritage faced stigmatization in the hospital. According to the nurse,^19^ “He (Caucasian male patient) stuck out his hand and then made a joke, ‘I probably shouldn’t shake your hand because you might have coronavirus.’ This was in front of a nurse, two medical students, and a few other people standing.”

As the virus spread to different countries, stories circulated that people of Asian origin were experiencing stigmatization and blame (Figure 2). High-profile people referred to the virus as the “Chinese virus” or “Wuhan virus.” The *Wall Street Journal* published an opinion column with a heading “China Is the Real Sick Man of Asia,” whereas other newspapers ran inappropriate headlines about Chinese students needing to stay away from school.^24^ There were reports of verbal and physical racial attacks against Chinese people in the earlier part of the pandemic. One Australian paper published a story with a headline “China kids stay home” that created miscommunication among the public, stigmatized the kids with Chinese heritage, and might cause a potential risk of discrimination at school.

There were multiple reports of physical harassment and violent attacks toward healthcare workers, people of Asian origins, people who were quarantined, or people who were evacuated from Wuhan. Our study identified 26 episodes of stigma related to violence. In Ukraine, local people blocked on the road and hurled stones at the buses that carried 82 passengers who were evacuated from Wuhan. An American citizen who was quarantined in the United States following evacuation from Wuhan shared his experience as,

They (community people) said you are infecting the whole of my country; you need to get the hell out.

There were also reports of self-stigma–associated death. For example, a man in India killed himself because of a misconception that he had coronavirus infection. The family members of the deceased mentioned that the person had a feeling of guilt and shame of contracting COVID-19 that he thought the virus would have unwittingly transmitted to family members along with an impression of how the society will react to that.

### Conspiracy theories.

Since the onset of the COVID-19 outbreak, several conspiracy theories had been circulating in China, Iran, Russia, United Kingdom, and the United States, and some of those were spread globally (Figure 3). One of the theories suggested that COVID-19 was a bioweapon and had been engineered by international agencies (Table 2). It is also argued that multiple countries manufactured and spread the deadly coronavirus in China as part of an economic and psychological war against China.^25^ On the other hand, people claimed that the virus was manufactured in a laboratory as China’s biowarfare program^26^ and that a scientist from China had engineered it as a weapon.^27^

Conspiracy theories have also emerged regarding the development of a COVID-19 vaccine or drug. One early theory postulated that a vaccine against this virus had already been invented, and this pandemic is an attempt to further vaccine sales.^28,29^ In the Middle East, a few government officials identified the pandemic as a conspiracy against the culture and honor of some religious cities in Iran.^30^ Another theory was circulating in social media that this pandemic is a population control scheme.^31^ During the public health crises, people often concentrate more on rumors and hoaxes than on science. The chief editor of Taiwan FactCheck Center said,^32^

Throughout this whole epidemic, people have liked conspiracy theories….Why are it that during epidemics, people don’t choose to believe accurate scientific information?

## DISCUSSION

Rumors, stigma, and conspiracy theories have the potential to decrease community trust in governments and international health agencies. Rumors can mask themselves as credible infection prevention and control strategies and have potentially serious implications if prioritized over evidence-based guidelines. For example, a popular myth that consumption of highly concentrated alcohol could disinfect the body and kill the virus was circulating in different parts of the world.^33^ Following this misinformation, approximately 800 people have died, whereas 5,876 have been hospitalized and 60 have developed complete blindness after drinking methanol as a cure of coronavirus.^34–37^ Similar rumors have been the reported cause of 30 deaths in Turkey.^38^ Likewise, in Qatar, two healthy South Asian men ingested either surface disinfectant or alcohol-based hand sanitizer after exposures to COVID-19 patients.^39^ In India, 12 people, including five children, became sick after drinking liquor made from toxic seed *Datura* (ummetta plant in local parlance) as a cure to coronavirus disease.^40^ The victims reportedly watched a video on social media that *Datura* seeds give immunity against COVID-19.^40^

Beyond individuals following misinformation, there have also been documented cases of organizations following inappropriate and misguided advice. A church in South Korea, where a spray bottle was used to spray salt water among the church attendees, resulted in more than 100 infections among the attendees because of spraying contaminated water. According to Lee Hee-young, head of Gyeonggi Province coronavirus task force in South Korea^41^: “It’s been confirmed that they put the nozzle of the spray bottle inside the mouth of a follower who was later confirmed as a patient before they did likewise for other followers as well, without disinfecting the sprayer.” Similar practices have been observed in other orthodox churches in the world.^42^ However, the number of infections that could be linked to the practice has not been documented.

Stigma and fears of discrimination might also have contributed to healthcare-associated infection in South Asia. People with COVID-19 may hide their symptoms or exposure histories when visiting hospitals, resulting in healthcare workers treating patients with minimum personal protective equipment that triggered healthcare-associated infections in Bangladesh.^43^ Because of the fear of stigma, people are also avoiding screening that may spread the deadly disease further.^44^ During this pandemic, there have been repeated accounts of verbal and physical abuse against people of Asian descent, and those involved in healthcare activities. Stigmatized people are vulnerable to social avoidance or rejection, poor health-seeking behavior, and physical violence.^8^ The stigma attached to COVID-19 patients, primarily related to the fear of contagion, has led to denying patient admission in hospitals in Uganda.^45^ The increasing number of COVID-19 cases, shortage of healthcare workers and resources, and their link with community transmission have resulted in violence not only against healthcare workers but also against healthcare facilities.^46^

Rumor, stigma, and conspiracy theories around public health emergencies are not new. During the initial days of the HIV epidemic,^47^ the rumor that HIV did not exist and its treatment was toxic to humans resulted in people refusing antiretroviral therapies in South Africa. Furthermore, the government promoted traditional medicines that fueled the vertical transmission of HIV in communities and cost >300,000 lives.^47^ Previous studies have also documented the conspiracy theories associated with the Zika virus, including that it was a biological weapon, which was circulating in the social media during the 2015–2016 outbreaks.^48^ Last, there are the well-documented issues with misinformation with Ebola, including false treatments, health workers were deliberately spreading the Ebola virus, and Ebola epidemic was a hoax.^49,50^ Such conspiracy theories and misinformation may have impeded the ability of healthcare workers and emergency responders to communicate with people about outbreak management and control measures.

The spread of rumors, stigma, and conspiracy theories not only affect the individuals but can also have consequences at the societal level, including the healthcare system. The rumor of complete lockdown had spread in several countries of the world that sparked panic-buying. The COVID-19 panic-buying drove up prices, and essential goods such as face masks, hand sanitizers, and toilet papers were out of reach for many people. Because of the extreme shortage of the supply of face masks and hand sanitizers, panic-buying may have contributed to hospital and home transmission of COVID-19 in several countries of the world.^51^

Prior research showed that public health interventions aimed at promoting evidence against misinformation quickly and clearly might change people’s perception and health-seeking behaviors.^52,53^ Currently, the WHO and other health agencies correct social media misinformation by defining it as myths.^54^ This approach has often been criticized for not including scientific evidence, ignoring context, and, therefore, less acceptable to the community people.^55^

This study has several limitations. First, this study focused on publicly available online platforms. Therefore, we did not follow and examine rumors and conspiracy theories circulating through other channels and offline. Second, for rumors, stigma, and conspiracy theories circulating in languages other than English, we relied on the use of Google Translate or an English version available at the fact-checking agency websites. Therefore, the findings may underestimate the actual prevalence of COVID-19 rumors, stigma, and conspiracy theories, and its impact on human health. Third, because of the rapid changes in information related to COVID-19, some myths can be misclassified as facts and vice versa. Therefore, the categorization of the infodemic may have been subject to misclassification bias. Finally, there may be variation in belief levels in the misinformation across countries. It was beyond the present study’s scope to determine the actual number of people who believed in the misinformation as fact.

Trust between healthcare workers and the affected community is essential to deal with the pandemic crisis. However, medical conspiracy theories can lead to mistrust with governments and health professionals that can impact people’s healthcare-seeking behavior,^56^ such as seeking out COVID-19 testing. Previously, it has also been shown that conspiracy theories can motivate people not to get vaccinated or receive antibiotics.^56^

In conclusion, misinformation fueled by rumors, stigma, and conspiracy theories can have potentially severe implications on public health if prioritized over scientific guidelines. Governments and other agencies must understand the patterns of COVID-19–related rumors, stigma, and conspiracy theories circulating the globe so that they can develop appropriate risk communication messages. Prior studies also found that people often visit international health agencies websites and the ministry of health’s sites for credible information. We recommend governments and international health agencies continue publishing correct and context-appropriate information supported by scientific evidence about COVID-19 on their websites. The national and international agencies, including the fact-checking agencies, should not only identify rumors and conspiracies theories and debunk them but should also engage social media companies to spread correct information.
